# Proteomic Landscape of Colorectal Cancer Derived Liver Metastasis Reveals Three Distinct Phenotypes With Specific Signaling and Enhanced Survival

**DOI:** 10.1016/j.mcpro.2025.101026

**Published:** 2025-07-04

**Authors:** Paula Nissen, Nadezhda V. Popova, Antonia Gocke, Daniel J. Smit, Geoffrey Yuet Mun Wong, Matthew J. McKay, Thomas J. Hugh, Kerstin David, Hartmut Juhl, Hannah Voß, Jens U. Marquardt, Björn Nashan, Hartmut Schlüter, Mark P. Molloy, Manfred Jücker

**Affiliations:** 1 Section Mass Spectrometric Proteomics, University Medical Center Hamburg-Eppendorf, Hamburg, Germany; 2 Institute of Biochemistry and Signal Transduction, University Medical Center Hamburg-Eppendorf, Hamburg, Germany; 3 Center of Molecular Neurobiology (ZNMH), University Medical Center Hamburg-Eppendorf, Hamburg, Germany; 4 Institute of Tumor Biology, University Medical Center Hamburg-Eppendorf, Hamburg, Germany; 5 Department of Upper Gastrointestinal Surgery, Royal North Shore Hospital, Sydney, NSW, Australia; 6 Bowel Cancer and Biomarker Research Laboratory, Faculty of Medicine and Health, School of Medical Sciences, The University of Sydney, Sydney, NSW, Australia; 7 Indivumed GmbH, Hamburg, Germany; 8 Department of Medicine I, University Medical Center, Lübeck, Germany; 9 Organ Transplantation Center, The First Affiliated Hospital of the University of Science and Technology of China, Hefei, China

**Keywords:** colorectal cancer, liver metastases, proteomics, signaling, alternative splicing, EpCAM, ECM, complement system, prognosis

## Abstract

Colorectal carcinoma is a major global disease with the second highest mortality rate among carcinomas. The liver is the most common site for metastases which portends a poor prognosis. Nonetheless, considerable heterogeneity of colorectal cancer liver metastases (CRC-LM) exists, evidenced by varied recurrence and survival patterns in patients undergoing curative-intent resection. Our understanding of the basis for this biological heterogeneity is limited. We investigated this by proteomic analysis of 152 CRC-LM obtained from three different medical centers in Germany and Australia using mass spectrometry-based differential quantitative proteomics. The proteomics data of the individual cohorts were harmonized through batch-effect correction algorithms to build a large multicenter cohort. Applying ConsensusClusterPlus to the proteome data yielded three distinct CRC-LM phenotypes (referred to as CRLM-SD (splice-driven), CRLM-CA (complement-associated), and CRLM-OM (oxidative metabolic)). The CRLM-SD phenotype showed higher abundance of key regulators of alternative splicing as well as extracellular matrix proteins commonly associated with tumor cell growth. The CRLM-CA phenotype was characterized by a higher abundance of proteins involved in the classical pathway part of the complement system including the membrane attack complex proteins and those with antithrombotic activity. The CRLM-OM phenotype showed higher abundance of proteins involved in various metabolic pathways including amino acids and fatty acids metabolism, which correlated in the literature with advanced proliferation of metastases and increased recurrence. Patients classified as CRLM-OM had a significantly lower overall survival in comparison to CRLM-CA patients. Finally, we identified a set of prognosis-associated biomarkers for each group including EpCAM, CEACAM1, CEACAM5, and CEACAM6 for CRLM-SD, DCN, TIMP3, and OLFM4 for CRLM-CA and FMO3, CES2 and AGXT for CRLM-OM. In summary, the discovery of three proteomic subgroups associated with distinct signaling pathways and survival of the CRC-LM patients provides a novel classification for risk stratification, prognosis and potentially novel therapeutic targets in CRC-LM.

## Highlights

• Liver metastases are common in colorectal cancer patients and are heterogeneous.
• Metastases heterogeneity is often neglected but important to specify treatment.
• Proteomics of a multicenter cohort revealed three major liver metastases subtypes.
• Proteomic liver metastases subtypes were associated with patient survival.
• Novel therapeutic targets recognizable by proteomic profiling of liver metastases.

## Graphical Abstract

**Figure:**
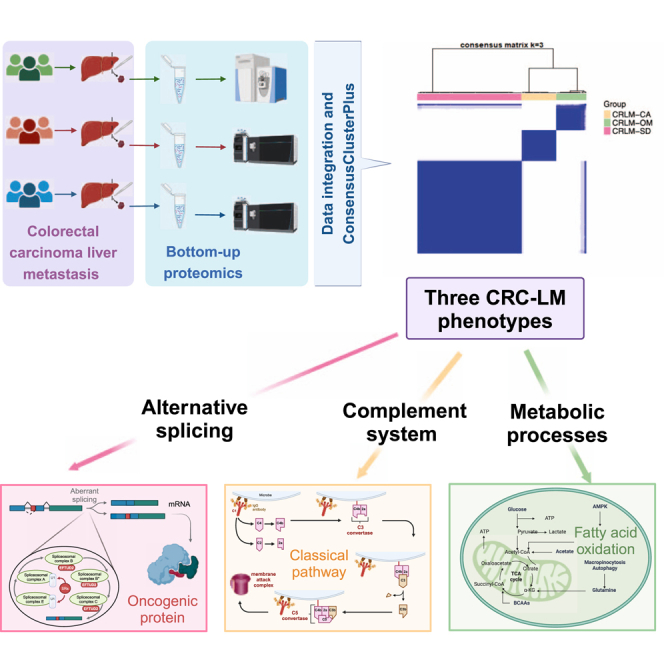

## In Brief

Proteomic analysis of a large muticenter cohort of colorectal carcinoma liver metastases samples revealed a differentiation into three proteomic phenotypes, independent of clinical parameters. The differentially regulated proteins and signaling pathways were associated with different prognostic features through literature mining. The findings from literature were consistent with the overall survival determined for each subgroup. The proposed biomarkers and enriched signaling pathways may enhance prognosis prediction and novel therapy development.

Colorectal cancer (CRC) is the fourth most common type of cancer worldwide and is among the prevalent causes of cancer related deaths (1, 2). About one-quarter of CRC patients will develop liver metastases (LM), which strongly impairs overall survival (OS) (3, 4, 5). Surgical resection is currently the most promising treatment but is often insufficient to eliminate recurrence (6, 7). Despite the curative intent of surgical resection, tumor cells might already have spread as circulating tumor cells or settled as disseminated tumor cells in distant organs (8). Therefore, early diagnosis is crucial to improve prognosis and survival in these patients (3).

A high degree of heterogeneity among colorectal cancers and the corresponding LM have been reported in the past (9, 10). Hence, it is important to define the different molecular patterns of CRC-LM to gain more knowledge of their biology that can be implemented in the clinical setting. Moreover, up to now it remains unclear which CRC patients will develop LM and why some patients develop more than one metachronous CRC-LM? In many cases, patients diagnosed with colorectal cancer can develop LM even several years after curative treatment and remission.

To elucidate the relevant proteins in CRC-LM, differential quantitative proteomics based on mass spectrometry opens new avenues due to a high number of detected proteins (11). In the previous work, we demonstrated a difference in the proteome profile between multiple metachronous metastases within the same patient analyzed through differential quantitative proteomics (12). The individual CRC-LM was characterized by a varying extracellular matrix (ECM) protein profile from first metachronous metastasis to the last one. To expand on this finding and characterize the proteomic landscape of synchronous and metachronous CRC-LM, we established a multicenter international cohort to analyze 152 fresh-frozen LM from 111 CRC patients.

## Experimental Procedures

### Experimental Design and Statistical Rationale

Fresh frozen liver metastasis tissue of CRC patients was retrieved from biobanks at three different centers (University Medical Center Hamburg-Eppendorf; University Hospital Schleswig-Holstein; Royal North Shore Hospital, Sydney, Australia) and was processed at different time points. Certified pathologists oversaw tumor dissection and selection for tumor enriched regions away from the surgical margin. The first cohort of samples was collected at the University Hospital Schleswig-Holstein (UKSH) in Lübeck, Germany and contained in total 42 LM from 42 CRC patients. The second cohort of LM tissue was collected at the University Medical Center Hamburg-Eppendorf (UKE) in Hamburg, Germany and stored by Indivumed GmbH. This cohort included 50 samples from 18 patients, although in four patients several samples from numerous metastases from different locations in the liver were collected. The third cohort was from the tumor bank of the Kolling Institute, Royal North Shore Hospital in Sydney, Australia and comprised 60 specimens collected from 51 patients. The first and second cohort were processed and measured by differential quantitative proteomics at the UKE in Germany whereas samples of the third cohort were processed and measured at the Kolling Institute in Australia. The raw MS data were collated by the UKE for a harmonized analysis. An overview of the cohorts is provided in Table 1 and more detailed in the Supplemental Table 1.

**Table 1:** Overview of liver metastasis samples from multiple medical centers

Cohort	Patients (n)	Metastases (n)	Synchronous	Metachronous	Metastases per patient
University Hospital Schleswig-Holstein (Lübeck, Germany)	42	42	7 (16.67%)	35 (83.33%)	42 × 1M
University Medical Center Hamburg-Eppendorf (Hamburg, Germany)	18	50	8 (16.00%)	42 (84.00%)	1 × 4M; 4 × 3M; 13 × 2M
Kolling Institute (Sydney, Australia)	51	60	23 (38.33%)	37 (61.67%)	1 × 3M; 7 × 2M; 43 × 1M

### Ethics Approvals

The study was conducted in accordance with the Helsinki declaration principles. Use of specimens and clinical data from the Kolling Institute, Sydney, was approved by the Northern Sydney Local Health District Human Research Ethics Committee (reference 2019/ETH12206). Written informed consent was obtained from each patient by the treating clinician before surgery. Use of the specimens from the University Medical Center Schleswig-Holsteins falls under the left-over tissue agreement from the University Mainz. The study including samples origin from the University Medical Center Hamburg-Eppendorf received approval by the competent ethics review committee of the medical association Hamburg under reference no. PV5035.

### Sample Preparation of CRC-LM (UKSH and UKE) for Bottom-Up Proteomics

Fresh frozen liver metastasis tissue was cut into smaller pieces, lysed through dilution in 0.1 M triethylammonium bicarbonate buffer (TEAB, Thermo Fisher Scientific) with 1% w/w sodium deoxycholate (Sigma-Aldrich), and afterward the tissue was homogenized using a TissueLyser (TissueLyser II, Qiagen). The sample was heated at 95 °C for 10 min for protein denaturation. To destroy the DNA/RNA, the lysates were sonicated five times at 30% intensity using a probe sonicator (Sonoplus 2200, Bandelin). Protein concentration was determined by bicinchoninic acid-assay according to the manufacturer`s instructions (Pierce BCA Protein Assay Kit, Thermo Fisher Scientific). Fifty micrograms protein was used for tryptic digestion. Disulfide bonds were reduced through incubation with 10 mM dithiothreitol (DTT, Sigma Aldrich) at 60 °C for 30 min followed by alkylation with 20 mM iodoacetamide (Sigma-Aldrich) for 30 min at 37 °C in the dark. For digestion of proteins, trypsin (sequencing grade, Promega) was added in a 1:50 ratio (enzyme to protein) and incubated overnight at 37 °C. Sodium deoxycholate was precipitated using 1% formic acid (FA, Fluka) with subsequent centrifugation for 10 min at 16,000g. The supernatant was transferred into a new tube and lyophilized using a SpeedVac vacuum concentrator (Thermo Fisher Scientific). The dried peptides were stored at −20 °C until further use.

### Sample Preparation of CRC-LM (Sydney) for Bottom-Up Proteomics

Tissue (approx. 100 mg) was first diluted in 400 μl lysis buffer containing of 150 mM potassium chloride, 2 mM EDTA, 100 mM TEAB, and 5 mM DTT and afterward lysed by using a TissueLyser (Qiagen, TissueLyser) for 8 min. Subsequently, 200 μl aliquots were taken and diluted with 200 μl 10% sodium dodecyl sulfate with 50 mM TEAB. Next, the diluted samples were heated at 100 °C for 15 min. The samples were centrifuged at 5 °C and 21,000*g* for 10 min, and the supernatant was collected. The protein concentration was determined by absorbance measurement at 280 nm using the Nanodrop One/Onec (Thermo Fisher Scientific).

To alkylate the remaining free cysteine thiol groups 8 μl of 500 mM iodoacetamide was added, and the samples were incubated for 10 min at room temperature. To prepare the proteins for tryptic digestion, they were bound to an S-Trap column (ProtiFi) and processed following the manufacturer’s protocol. Following overnight digestion through incubation with trypsin (sequencing grade, Promega) in a 1:20 trypsin-to-protein ratio at 37 °C, peptides were eluted sequentially using 50 mM TEAB buffer, then 0.2% FA in water, then 50% acetonitrile (ACN) in water. The eluted peptide solutions were combined and dried in a vacuum centrifuge.

### Differential Quantitative Proteomics in DDA Mode

The UKSH and UKE cohorts were measured in data-dependent acquisition (DDA) mode as described below. Samples were measured in a randomized order.

Prior to mass spectrometric measurement, the peptide pellets were each resuspended in 20 μl of 0.1% FA to obtain a final concentration of this of 1 μg/μl. Differential quantitative proteomics measurement of the samples was performed on a quadrupole-ion trap-orbitrap mass spectrometer (Orbitrap Fusion, Thermo Fisher Scientific) coupled to a nano-ultra-high-pressure liquid chromatography (UPLC) (Dionex UltiMate 3000 UPLC system, Thermo Fisher Scientific). One microliter of each peptide sample was injected into the chromatographic system via an autosampler. Subsequently, the injected samples were purified and desalted through a reversed-phase trap column (Acclaim PepMap 100 C18 trap; 100 μm × 200 mm, 100 Å pore size, 5 μm particle size; Thermo Fisher Scientific) and transferred to a C18 reversed-phase column (Peptide BEH C18, 75 μm × 250 mm, 130 Å pore size, 1.7 μm particle size; Waters) for chromatographic separation. Initial chromatographic purification over the trap column was performed for 5 minutes at a flow rate of 15 μl/min using 99% solution A (0.1% FA in water) and 1% solution B (0.1% FA in ACN). For separation and elution of the peptides, a linear 80 min gradient of 1 to 30% of solution B was applied within 70 min at a flow rate of 0.3 μl/min. Ionization of the eluted peptides was performed using a nano electrospray ionization source (nano ESI) with a spray voltage of 1800 V. The ionized peptides were passed into the mass spectrometer and analyzed in DDA mode. For each MS1 scan, ions were accumulated for a maximum of 120 milliseconds or until a charge density of 2 x 10^5^ ions (automatic gain control, (AGC) target) was reached. Fourier-transformation-based mass analysis of the data from the orbitrap mass analyzer was performed in a covered mass range of 400 to 1300 m/z with a resolution of 120,000 at m/z = 200. Further, peptides from each precursor scan with a charge number between 2+ and 5+, and above an intensity of 1000 were isolated within a 1.6 m/z isolation window in Top Speed mode for 3 seconds and fragmented with a normalized collision energy of 30% using higher energy collision induced dissociation. MS2 scans were generated using an ion trap mass analyzer at a rapid scan rate, covering a mass range starting at 120 m/z and accumulated for 60 ms or up to an AGC target of 1 x 10^5^. Dynamic exclusion of all fragmented peptides was performed for 30 s after precursor selection.

### Differential Quantitative Proteomics in DIA Mode

The Sydney cohort was acquired in data-independent acquisition (DIA) mode as described below. Samples were measured in a randomized order.

Prior to mass spectrometric measurement, the peptide pellets were each resuspended to a final concentration of 0.4 μg/μl in 0.1% FA. The concentration was verified by absorbance measurement at 280 nm using the Nanodrop One (Thermo Fisher Scientific). Differential quantitative proteomics measurement of the samples was performed in a quadrupole Orbitrap mass spectrometer (QExactive HF-X Hybrid Quadrupole-Orbitrap mass spectrometer, Thermo Fisher Scientific) coupled to a nano UPLC (Dionex UltiMate 3000 UPLC system, Thermo Fisher Scientific). Five microliters of each peptide sample was injected into a chromatographic column via an autosampler. The injected samples were transferred to a self-packed C18 reversed-phase analytic column for chromatographic separation (35 cm × 75 μm ID; 1.9 μm beads; Dr Maisch). The column temperature was kept at 60 °C. Subsequently to separation, the sample was loaded onto the column for 20 min at 5% solution B (80% ACN and 0.1% FA). For separation and elution of the peptides, a linear gradient of 5 to 40% of solution B was applied within 100 min at a flow rate of 0.3 μl/min. Then the amount of solution B was increased to 95% in 5 min and holding there for 5 min. Afterward, solution B was decreased again to 5% in 1 min and stayed there for 9 min. The total run time resulted in 140 min. Ionization of the eluted peptides was now performed using a nano-electrospray ionization source (nano ESI) with a spray voltage of 1800 V. The ionized peptides were separated from the solution A by a nano ESI. The ionized peptides were passed into the mass spectrometer and analyzed in DIA mode. For each MS1 scan, ions were accumulated for a maximum of 55 ms or until a charge density of 3 x 10^6^ ions (AGC target) was reached. MS1 spectra were acquired with a resolution of 30,000 within a mass-to-charge range of 395 to 1005 m/z. Further, peptides from each precursor scan were isolated within a 12 m/z isolation window and fragmented with a normalized collision energy of 27% using higher energy collision induced dissociation. MS2 scans were generated using an Orbitrap mass analyzer with a dynamic first mass and an Orbitrap resolution of 15,000 and a cumulation of 25 ms or up to an AGC target of 1 x 10^6^. The number of data acquisition cycles was set to 50.

### DDA Data Processing

Liquid chromatography tandem mass spectrometry (LC-MS/MS) raw data were searched, and peak lists were generated with the Sequest HT algorithm integrated in Proteome Discoverer software (v 2.4.1.13, Thermo Fisher Scientific) against a reviewed *Homo sapiens* Swiss-Prot database, obtained in April 2020, containing 20,365 entries. Carbamidomethylation of cysteine ends was set as fixed modification and methionine oxidation as well as pyroglutamate formation at glutamine residues at the peptide N terminus as variable modification. All peptides in a size range of six to 144 amino acids were identified with a precursor mass tolerance of 10 ppm and a fragment mass tolerance of 0.6 Da. Only peaks exceeding a signal-to-noise ratio of 1.5 were used for identification and quantification. Only peptides with a maximum of two missed tryptic cleavages were considered. Furthermore, matching between runs with a hit time window of 0.2 min was used to identify peptides. For peptide and protein identification, a strict false discovery rate (FDR) value limit of <0.01 were used. Label-free quantification using the Minora Algorithm implemented in Proteome Discoverer was performed. Protein abundances were calculated by summing sample abundances of connected peptide groups from at least three distinct peptides through the top-N approach. Precursor abundances were calculated based on the intensity.

### DIA Data Processing

Raw data from LC-MS/MS measurements were processed by DIA-NN version 1.8.1 (13) using the library-free mode. Before processing, the.raw files were demultiplexed and transformed to.mzml files using MSconvert by ProteoWizard (version 3.0.19066–20fa73629). For library-free mode, trypsin was selected as protease, the FASTA-digest for library generation was enabled as well as Deep-learning based spectra, retention time, and ion mobility prediction. This way DIA-NN created, prior to protein identification, an *in-silico* library out of all measured retention times, m/z-values and associated MS2 spectra by matching those to the *Homo sapiens* Uniprot FASTA file downloaded 2019, containing 20,409 entries. For protein identification the generated library was matched to all MS1 and related MS2 spectra of tryptic digested proteins.

Carbamidomethylation of cysteine residues was reported as a fixed modification. Methionine oxidation and acetylation of the protein N terminus were indicated as variable modifications. The maximum number of variable modifications was set to three. All peptides within a size range of seven to 30 amino acids, with a precursor charge range of one to four, a precursor m/z range of 300 to 1800 and a fragment ion m/z range of 200 to 1800 were considered for identification. Only peptides with a maximum of two missed tryptic cleavages were considered. Mass accuracy, MS1 accuracy, and scan window were set automatically based on the first run (default) to 15 ppm for mass accuracy, 17 ppm for MS1 accuracy, and 7 for scan window radius. Furthermore, matching between runs was used to identify peptides. For peptide identification, an FDR value limit of <0.01 and a reverse approach for decoy peptide databases were used. For label-free quantification, all identified razor and unique peptides were observed.

### Statistical Rationale

For quantitative proteomics statistical analysis the relative protein abundances from each cohort were separately loaded into Perseus software (Max Plank Institute for Biochemistry, v 1.6.15.0) separately, log2 transformed and column median normalized at the protein level to correct possible slight variations in peptide amounts injected (14). Raw data for each cohort including protein identification and quantification information are listed in Supplemental Table S2–4. For those proteins identified based on < 2 unique peptides which remained in the valid values filtered data, annotated spectra data have been deposited to the ProteomeXchange Consortium via the PRIDE partner repository with the dataset identifier PXD055821. The transformed and normalized data were taken for batch-effect correction in R software environment (version 4.3.1) using the HarmonizR framework (version 0.0.0.9000) (15). The batch effect of the processing batches (three cohorts) was removed applying the ComBat method (mode 1) due to Gaussian distribution of protein abundances.

To determine the ideal number of clusters within the batch-effect corrected normalized data an unsupervised consensus clustering was conducted using the ConsensusClusterPlus package (version 1.64.0) in the R software environment (16, 17). The data were tested with two clustering methods (k-means and hierarchical clustering) for 1000 repetitions. As k-means works with Euclidean distance, only one distance metric was tested. The Ward’s D method was used for inner and final linkage. The proportion of ambiguous clustering score was calculated manually and plotted using the ggplot2 package (version 3.4.4) (18). As the k-means and hierarchical clustering both showed the strongest minimum at k = 3, this clustering was verified by NIPALS-based principal component analysis (PCA) and Uniform Manifold Approximation and Projection for dimension reduction (UMAP). The cluster annotation from k-means clustering was used for further analysis as it showed the best cluster formation. The three subgroups are referred to as CRLM-SD (splice-driven), CRLM-CA (complement-associated) and CRLM-OM (oxidative metabolic) hereafter. The protein abundances after batch effect reduction and with samples assigned to the subgroups are depicted in Supplemental Table S5.

The data were filtered for proteins present in at least 70% of all samples. Heatmap visualizations of hierarchical clustering, NIPALS-PCA, UMAP, and box plots were created using R software environment. The NIPALS-based principal component calculation and visualization was conducted using the mixOmics package (version 6.24.0) in Bioconductor (version 3.18) (19). Dimensionality reduction and visualization was performed using the umap package (version 0.2.10.0) (20). Hierarchical clustering was performed in the ComplexHeatmap package (version 2.16.0) using the Pearson distance metric and a ward.D linkage (21, 22). For ANOVA multiple sample test all proteins were considered significant with a permutation-based FDR value of <0.05.

Pathway enrichment analysis was performed visualizing gene set enrichment analysis (GSEA, version 4.2.3) results of each group tested against the other groups using Cytoscape (version 3.10.0) together with EnrichmentMap according to the protocol of J. Reimand and R. Isserlin *et al* (23, 24, 25). For GSEA the gene ontology gene sets derived from the Molecular Signatures Database Human Collections (v.2023.2) were used (26, 27). The node cutoff was set to q-value <0.1 and the edge cutoff to q-value <0.5089. Only gene sets which belong to an AutoAnnotation set with at least five, for CRLM-SD versus REST and CRLM-CA versus REST, and at least six gene sets for CRLM-OM versus REST were displayed.

Signaling pathway enrichment analysis for the Student’s *t* test significant (Benjamini–Hochberg FDR <0.05, two-fold change) differentially abundant proteins from each test comparing one subgroup against all others was performed with the ingenuity pathway analysis (IPA) software (version 94302991, Qiagen IPA, Qiagen) (28). Benjamini-Hochberg FDR was chosen due to the large cohort size and the normally distributed data. For visualization of canonical pathways and networks overrepresented in one subgroup, all pathways, and networks of proteins across the entire QIAGEN Knowledge Base were tested using a one-sided, right tailed Fisher’s exact test (statistical significance was set at FDR-corrected *p* < 0.05). The match between expected and observed upregulation and downregulation patterns was quantified using a z-score.

For the Kaplan–Meier curve analysis, the survival was set as the difference between the diagnosis (or referral for Sydney cohort) of the individual LM and the date of death or the last check-up if the patient is still alive.

**Fig. 1:**
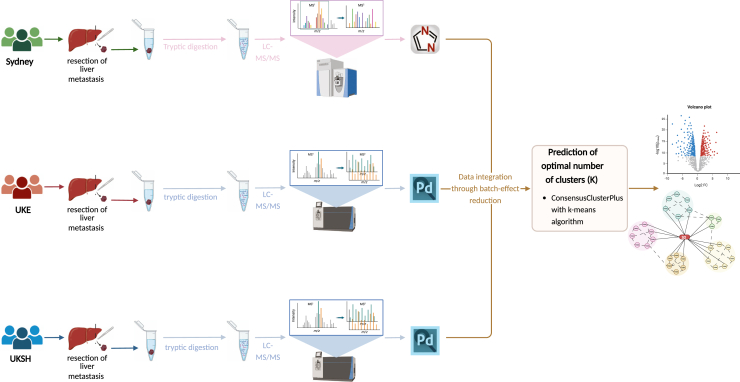
**Proteomics workflow of international CRC-LM cohort**. Workflow from CRC-LM sample retrieval in three individual studies to LC-MS/MS data integration through batch-effect reduction and subsequent bioinformatical analysis toward definition of individual molecular signatures of three proteomic phenotypes. The fresh frozen tissue slices were homogenized, lysed and the proteins were cleaved into peptides through tryptic digestion. Tryptic peptides were analyzed using LC-MS/MS and proteins were identified and quantified using database search software (DIA-NN for Sydney cohort and Proteome Discoverer for UKE and UKSH cohort). Created in https://BioRender.com. CRC-LM, colorectal cancer liver metastases; LC-MS/MS, liquid chromatography tandem mass spectrometry.

Literature mining was performed using an in-house pipeline adapted from the OmixLitMiner tool (29, 30). Graphical abstract, Figure 1 and Figure 4*A* was created in BioRender. Nissen, P. (2025) https://BioRender.com/i92f876, https://BioRender.com/i02h369 and https://BioRender.com/tnrtbfo.

**Fig. 2:**
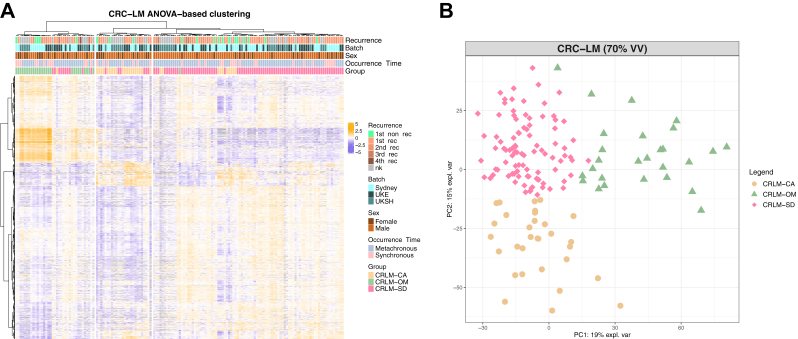
**Three CRC-LM phenotypes revealed through ConsensusClusterPlus**. ConsensusClusterPlus algorithm revealed a statistical optimal cluster number of three, visualized through (*A*) heatmap visualization of hierarchal clustering of all 1667 ANOVA significant (q-value<0.05) proteins identified in 70% of all samples in the international cohort after batch-effect reduction. The (*B*) principal component analysis of all proteins identified in 70% of all samples is depicted as a scatter plot.

**Fig. 3:**
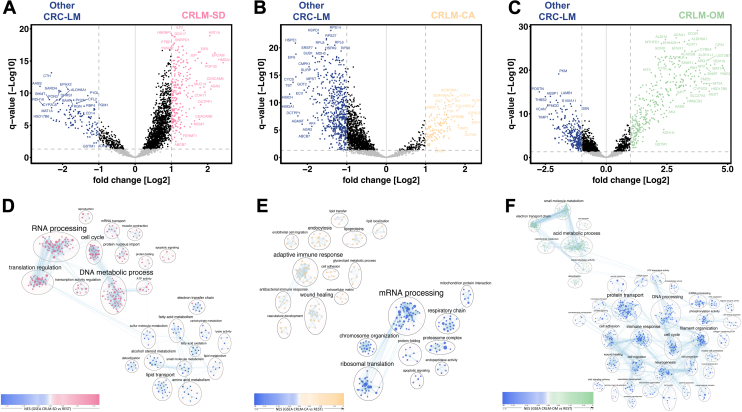
**Differential abundant proteins and associated signaling pathways between CRC-LM subgroups**. Volcano Plots (*A*–*C*) with all proteins plotted according to their assigned fold change and q-value in the *t* test of (*A*) CRLM-SD against the rest, (*B*) CRLM-CA against the rest and (*C*) CRLM-OM against the rest. All proteins considered significant abundant (q-value <0.05) are marked in *blue* (fold change ≤2) and in Group color (fold change ≥2). EnrichmentMap based clustering of gene set enrichment analysis (GSEA) derived gene sets enriched in (*D*) CRLM-SD, (*E*) CRLM-CA and (*F*) CRLM-OM visualized using Cytoscape. For each GSEA analysis one group was tested against all other groups. Encircling of the gene sets was created with the AutoAnnotate function in Cytoscape. CRLM-SD, CRLM-splice-driven; CRLM-CA, CRLM-complement-associated, CRLM-OM, CRLM-oxidative metabolic.

**Fig. 4:**
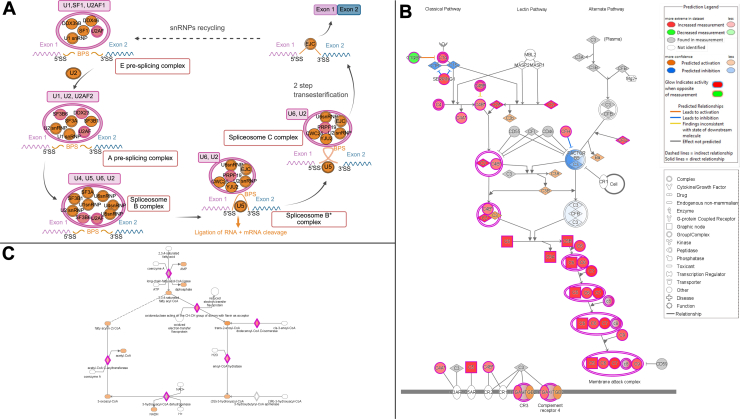
**IPA pathways specific to each CRC-LM phenotype**. Canonical pathways extracted from ingenuity pathway analysis (IPA) for (*A*) CRLM-SD, (*B*) CRLM-CA, and (*C*) CRLM-OM. For CRLM-SD the adapted spliceosomal cycle is depicted in (*A*). For CRLM-CA the complement system pathway is projected in (*B*) and for CRLM-OM the fatty acid beta oxidation I in (*C*). All *red* marked proteins (complexes) were found significantly higher abundant in the assigned group (q-value <0.05; 2 FC). All *green* marked proteins (complexes) were found significantly lower abundant in the assigned group (q-value <0.05; 2 FC). Gray proteins were identified in the dataset but not significantly abundant. *White* proteins were not found in the dataset. *Orange* and *blue* proteins were predicted to be increased or decreased in concentration based on linked significantly abundant proteins found in the pathway. CRLM-SD, CRLM-splice-driven; CRLM-CA, CRLM-complement-associated; CRLM-OM, CRLM-oxidative metabolic; FC, fold change.

### Immunohistochemistry and Clinical Data

Immunohistochemistry (IHC) was performed using 4 μm sections from formalin-fixed paraffin-embedded blocks containing primary CRC and matched CRC-LM using monoclonal anti-EpCAM antibody (#93790, Cell Signaling Technology) and anti-Clusterin antibody (#34642, Cell Signaling) in 1:400 dilution. Visualization was achieved using diaminobenzidine chromogen. Signal localization was confirmed by an accredited anatomical pathologist.

Mismatch repair status and *KRAS* mutations were retrieved from patient pathology reports where available. Mismatch repair was assessed by IHC for loss of MLH1, MLH2, MLH6, and PMS2. *KRAS* mutations were assessed through next-generation sequencing.

## Results

This study analyzed individual patient cohorts of macrodissected fresh frozen CRC-LM tissues obtained from two centers in Germany and one from Australia. These cohorts are referred to as UKSH cohort, UKE cohort, and Sydney cohort. Each cohort was independently measured by proteomic mass spectrometry (Fig. 1).

After integration of all three cohorts through batch-effect correction 9506 proteins were identified in the total cohort. A total of 7809 proteins were found in at least three samples in each subgroup and 3044 were found in 70% of all samples (Supplemental Table S6).

### Proteome Analysis of the Multicenter CRC-LM Cohort Revealed Three Proteomic Phenotypes

Based on our previous study on the differentiation within three LM from one patient developed at different time points, we aimed to expand on this concept and characterize the different molecular patterns of LM from colorectal cancer patients (12). To compare CRC-LM proteomes, we determined the mathematical most probable number of clusters within the cohort using consensus clustering with k-means and hierarchical clustering algorithms. Both clustering methods demonstrated three groups to be the statistical optimal number of clusters within the dataset (Supplemental Fig. S1).

The classification into three clusters by k-means resulted in 94 samples in cluster group one, 31 samples in cluster group two, and 27 samples in cluster group three. The clustering into the three groups, in following referred to as CRLM-SD, CRLM-CA, and CRLM-OM, was supported additionally through Pearson-correlation-based hierarchical clustering of all 1667 ANOVA multiple-sample testing significant (q-value <0.05) proteins (Fig. 2*A*) and the NIPALS-based PCA, created with the mixOmics package in R, of all proteins present in at least 70% of all samples (Fig. 2*B*).

Importantly, we observed that the hierarchical clustering was independent from clinical parameters, including sex, timing of occurrence relative to the primary tumor and recurrence, or batch effects. To reassure the formation of these distinct subgroups was not based on sample cohort, same patients of origin, run order of the samples, time of occurrence of the LM or regime of pretreatment additional PCA, (Supplemental Fig. S2) and hierarchical clustering (Supplemental Fig. S3) were performed with that information annotated. Furthermore, we investigated for any bias of the phenotype formation due to contamination of the tissue samples with blood. The unsupervised analysis of the normalized protein abundances of the most abundant blood proteins showed no differentiation into the three phenotypes (Supplemental Fig. S4) (31, 32), suggesting that blood contamination was not a driver of the LM phenotype.

Together, this analysis confirmed that the differences between the clusters were based on quantitative differences in the tumor proteome and were independent of clinical or technical parameters.

### Differentiation Into Subgroups Is Based on Distinct Molecular Signatures Assigned to Each Subgroup

The interpretation of each individual cluster was accomplished through statistical analysis and by GSEA to outline the dominant proteins and gene sets. Thereby we aimed to determine the prevalent signaling pathways in each phenotype. The analysis resulted in 351 significantly different abundant proteins when comparing CRLM-SD against the rest, 713 proteins when testing CRLM-CA against the rest and 612 in the comparison of CRLM-OM against the rest (Fig. 3, *A–C*).

Derived from GSEA, many of the significantly high abundance proteins, observed in CRLM-SD compared to the other groups, were involved in translation and transcription processes (Fig. 3*D*). More precisely, proteins assigned to RNA processing associated gene sets, like SRSF7, hnRNPA1, PTBP1, hnRNPL, and PHF5A as transregulatory factor splicing linked proteins, were found in higher abundance. The high normalized enrichment score of the ribonucleoprotein complex gene set showed a significant higher abundance of ribosomal and ribonucleoproteins (Supplemental Table S7).

The main features of CRLM-SD were further analyzed by IPA (see all enriched terms in Supplemental Table S8). As part of the transcription process, the processing of capped intron-containing pre-mRNA as well as the spliceosomal cycle was significantly enriched in CRLM-SD compared to the other subgroups (Fig. 4*A*). The depicted pathway orthogonal validated an enrichment in proteins involved in various splicing processes, especially alternative splicing. Overall, the main proteomic feature of subgroup CRLM-SD was the higher abundance of translation and transcription correlated proteins.

The GSEA of subgroup CRLM-CA in comparison to all other groups revealed the strongest enrichment in gene sets associated with immune response related processes and signaling pathways (Fig. 3*E*). Part of this gene sets and the gene ontology biological pathway (GOBP) complement activation set are complement factors including C2, C4A, C5, C6, C7, C8A, C8G, and C9 (Fig. 3*B*).

This finding was analyzed in detail using IPA. In the visualization of the enriched complement system with the main three pathways depicted, the classical complement pathway seems to be the dominant one in subgroup CRLM-CA compared to the other CRC-LM subgroups (Fig. 4*B*). Particularly, all proteins part of the membrane attack complex (MAC) were found to be significantly higher abundant in CRLM-CA. These included the proteins C5, C6, C7, C8, and C9, which were all found within the significantly high abundance proteins. To validate the LC-MS/MS detected findings of complement cascade components, IHC of clusterin as representative protein was performed in specimens of each of the CRC-LM subgroups (Supplemental Fig. S5). The increased expression of clusterin in CRLM-CA-type LM and its low detection in the remaining subgroups was in line with the mass spectrometric data. We noted the increased expression was mostly visible in the hepatocytes surrounding the epithelial tumor cells in CRLM-CA specimens, suggesting an interaction of the CRLM with the tumor microenvironment. Closely related to the reactions of the immune system in the context of cancer are alterations in glycosylation of proteins, wherefore all *t* test significantly high abundance proteins in CRLM-CA were searched against a human liver-specific glycoprotein database containing 2483 glycoproteins (GlycositeAtlas, updated 2016) (33). Of all 130 high abundance proteins, 85 were identified as glycoproteins which is typical of the cell surface and secreted proteins (Supplemental Table S9).

Another collection of gene sets found abundant in CRLM-CA are those of the ECM. Decorin was one of those ECM proteins, which is also known to be a tumor suppressor in CRC (34, 35, 36). It was found among the *t* test significant proteins with the 20 highest difference values and was detected in all samples (Fig. 3*B* and Fig. 6*B*).

**Fig. 5:**
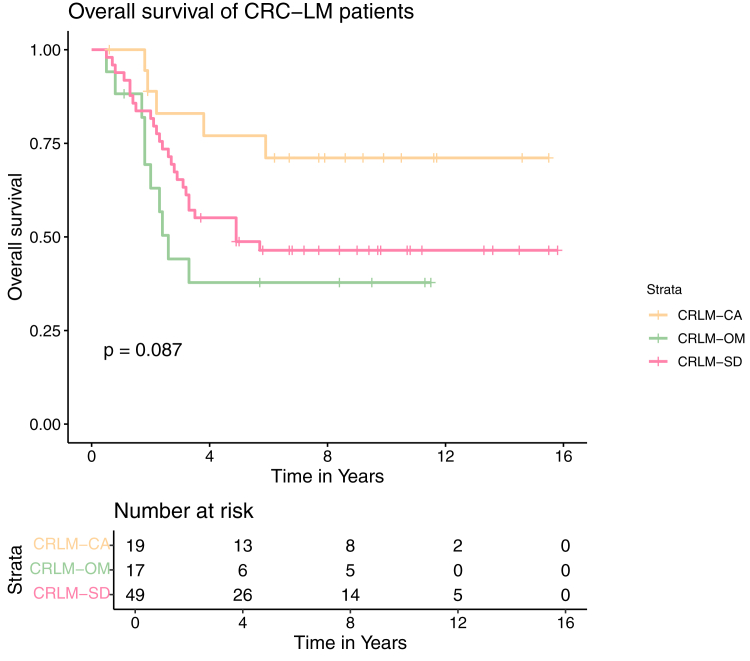
**Kaplan–Meier curve and risk table of overall survival time of patients with CRC-LM assigned to phenotype CRLM-SD, CRLM-CA, or CRLM-OM**. The *p*-value depicted in the plot referred to the survival data difference of the displayed groups. CRC-LM, colorectal cancer liver metastases; CRLM-CA, CRLM-complement-associated; CRLM-OM, CRLM-oxidative metabolic; CRLM-SD, CRLM-splice-driven.

**Fig. 6:**
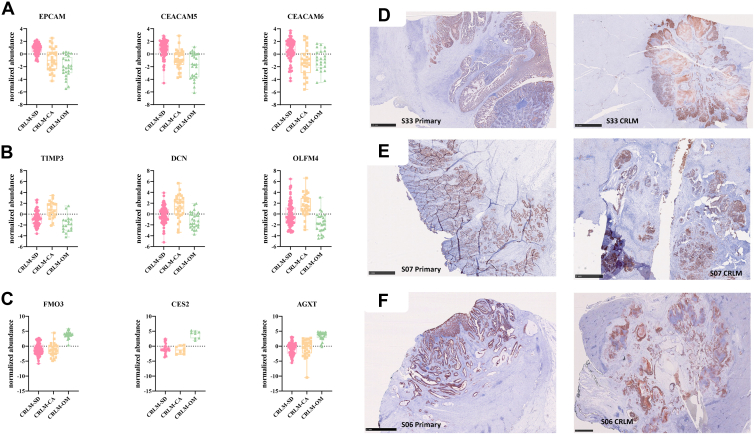
**Biomarker panel specific to each CRC-LM subgroup**. Box plot visualization of proteins which are significant high abundant in (*A*) CRLM-SD, (*B*) CRLM-CA and (*C*) CRLM-OM. The log2-transformed, column median normalized abundance values were used. Representative immunohistochemistry (IHC) staining of EpCAM in primary tumor tissue and corresponding liver metastases of three patients from the Sydney cohort. The CRC-LM from (*D* and *E*) were assigned to the proteomic subgroup CRLM-SD and (*F*) to CRLM-CA. EpCAM was only present in the tumor cells and not in hepatocytes. CRC-LM, colorectal cancer liver metastases; EpCAM, epithelial cell adhesion molecule; CRLM-SD, CRLM-splice-driven; CRLM-CA, CRLM-complement-associated, CRLM-OM, CRLM-oxidative metabolic.

A higher abundance of ECM proteins was identified as a feature which CRLM-SD and CRLM-CA had in common, although it is more pronounced in CRLM-CA. In CRLM-SD, 15 ECM proteins were found enriched, whereas CRLM-CA displayed 47 ECM proteins. The higher abundance of ECM proteins in CRLM-SD and CRLM-CA is a key characteristic, which distinguishes them from CRLM-OM.

Based on the GSEA of cluster CRLM-OM against the other subgroups, CRLM-OM is strongly characterized by high abundance of proteins related to liver-specific metabolism pathways. Especially, gene sets linked to acid metabolic processes and carbohydrate metabolism pathways were enriched. As part of the acid metabolism, proteins annotated to the amino acid metabolic process and fatty acid metabolic process gene sets were found among the proteins with the highest difference values.

IPA confirmed the upregulation of various metabolism pathways in CRLM-OM. One of those with the lowest *p*-value was the fatty acid beta oxidation I with the main regulatory enzymes significantly higher abundant in CRLM-OM (Fig. 4*C*). Therefore, subgroup CRLM-OM was characterized mainly by liver specific amino acid and fatty acid metabolism pathways.

### CRC-LM Subgroups Correlated With Different OS

To underline the findings from enrichment analysis and literature mining, survival (OS) data were integrated through Kaplan–Meier analysis (Fig. 5). The Kaplan–Meier curves reflected the better OS of patients after being diagnosed with LM assigned to CRLM-CA in comparison to the other CRC-LM subgroups. A significant OS difference was confirmed between CRLM-CA and CRLM-OM patients (*p* = 0.03). This enhanced OS of CRLM-CA-type patients is in line with the significantly higher abundant ECM proteins and the classical complement cascade enriched in CRLM-CA which is associated with a better survival.

### Identification of Prognosis-Associated Biomarkers of Each CRC-LM Subgroup

For diagnosis of the specific CRC-LM subtype in the clinic, the list of significantly high abundance proteins in each subgroup was screened for potential biomarkers applying the following criteria: the potential biomarker should be among the proteins with the highest difference, detectable in body fluids like blood, feces, urine, or saliva and reflect the CRC-LM subgroup specific survival. Literature mining of all differentiated proteins was performed with an in-house pipeline.

As a result, the following proteins were selected as potential biomarkers for the individual CRC-LM subgroups according to the above-named criteria (Fig. 6, *A–C*).

To identify a liver metastasis assigned to CRLM-SD, epithelial cell adhesion molecule (EpCAM), carcinoembryonic antigen-related cell adhesion molecule 5 and 6 (CEACAM5 and CEACAM6) were proposed (Fig. 6*A*). As CEACAM family members are of interest in CRC context and can be detected in blood or fecal patient samples, elevated CEACAM proteins (relative to CRLM-CA/CRLM-OM) were proposed as diagnostic markers for subtype CRLM-SD LM (37, 38, 39).

The third proposed biomarker for CRLM-SD-type CRC-LM is EpCAM, which is already a known biomarker for CRC associated with poorer prognosis (40, 41). The presence of EpCAM in LM assigned to CRLM-SD was validated through IHC with anti-EpCAM antibody in fresh frozen LM tissue classified as CRLM-SD from two patients of the Sydney cohort (Fig. 6, *D* and *E*). The EpCAM staining of the corresponding primaries of each patient revealed the presence of EpCAM already in the primary tumor.

Biomarkers, which could yield a clinical identification of the CRLM-CA subtype patients, are metalloproteinase inhibitor 3 (tissue inhibitor of metalloproteinases 3; TIMP3), decorin (DCN), and olfactomedin-4 (OLFM4) (Fig. 6*B*). All three proteins are glycoproteins and associated with immunology pathways in cancer (42, 43, 44).

To distinguish CRC-LM subgroup CRLM-OM from the others in clinical diagnostic, flavin-containing monooxygenase 3 (FMO3), cocaine esterase (CES2) and alanine-glyoxylate aminotransferase (AGXT) may be of potential use (Fig. 6*C*).

Altogether, we detected three different distinct CRC-LM phenotypes based on their proteome profile, which correlated with different OS in a multicenter study.

## Discussion

The investigation of the proteome is of central importance to understand the biological processes leading to CRC-LM and the development of metachronous LM during the course of the disease. This fundamental research is needed to identify distinct CRC-LM types in different patients to adapt the treatment for the different survival groups. To our knowledge, this is the largest proteomic study to investigate features of CRC-LM. Our multicenter investigation of the proteome profiles of 152 CRC-LM samples from 111 patients uncovered, for the first time, distinct subgroups of CRC-LM associated with specific signaling and survival of the patients.

Each of the three subgroups i.e. CRLM-SD, CRLM-CA, and CRLM-OM, showed a specific proteome profile with enrichment of specific signaling pathways. Important to highlight is that the subgroup formation was not biased by clinical parameters and is clearly a lesion feature rather than a patient feature as seen in Supplemental Figure S3, *A* and *B*. This was occasionally observed for metachronous lesions, which is consistent with independent evolution of tumor subclones.

The associated prognosis of the significantly high abundance proteins and enriched signaling pathways identified in each subgroup compared to the other ones, are in line with the OS found for each CRC-LM subgroup, as summarized in Table 2.

**Table 2:** Overview of significantly high abundance proteins in each CRC-LM phenotype and their prognosis stated in the literature

CRLM-SD		CRLM-CA		CRLM-OM	
Protein	Prognosis	Protein	Prognosis	Protein	Prognosis
VCAN (45)	down	TIMP3 (46)	up	CPT2 (47)	down^a^
LAMC1 (48)	down	DCN (35)	up	ACSL1 (49)	down
SRSF7 (50)	down	C1q (51)	up	FMO3 (52)	down^a^
hnRNPL (53, 54, 55)	down	SERPINF1 (56)	up	CES2 (57)	down
hnRNPA1 (50)	down	OLFM4 (59)	up	AGXT (58)	down
PTBP1 (50)	down				
PHF5A (60)	down				
CEACAM1 (53, 54, 55)	down				
CEACAM5 (61, 62)	down				
CEACAM6 (61, 62)	down				
EpCAM (41, 63, 64, 65)	down				

ACSL1, long-chain-fatty-acid-CoA ligase 1; AGXT, aminotransferase; CES2, cocaine esterase; CPT2, carnitine acyltransferase II; FMO3, flavin monooxygenase 3; hnRNPL, heterogeneous nuclear ribonucleoprotein L; LAMs, laminins; SRSF, serine/arginine-rich splicing factor; VCAN, versican; SERPINF1, serpin F1, also known as pigment epithelium-derived factor.

a Indirect through pathway.

One important molecular signature shared by CRLM-SD and CRLM-CA-type CRC-LM is the significantly higher abundance of ECM proteins compared to CRLM-OM, although this set of proteins was more dominant in CRLM-CA. Versican (VCAN) and LAMC1, which are strongly associated with tumor progression and increased metastasis risk (45, 48), were identified only in CRLM-SD, whereas TIMP3 and decorin, which are linked to an opposing effect in cancer (36, 46), were found exclusively in CRLM-CA (Table 2). The increased abundance of ECM proteins was observed in our previous study on multiple metastases of one patient in the last metastasis developed before death (12). Moreover, we recently showed that VCAN is elevated in CRC-LM of patients with early intrahepatic recurrence after curative-intent resection of CRC-LM, further underscoring the prognostic significance of this protein (66).

A characteristic upregulation observed in the CRLM-SD-type CRC-LM was the higher abundance of proteins responsible for alternative splicing. The proteins elevated in CRLM-SD, which include five proteins i.e. SRSF7, hnRNPA1, PTBP1, hnRNPL, and PHF5A linked to trans-regulatory factor splicing suggest the involvement in alternative RNA splicing as a biological process of liver metastasis in this CRC-LM subgroup (Table 2) (53, 55, 60, 67, 68, 69). Furthermore, the significantly higher abundance of carcinoembryonic antigen proteins i.e. CEACAM1, CEACAM5, and CEACAM6 in CRLM-SD LM, which are associated with transforming growth factor-β pathway inhibition, and poor prognosis is in line with the lower survival in the CRLM-SD subgroup (61, 62).

EpCAM is another marker protein of clinical importance identified in the CRLM-SD-type CRC-LM. As it is only so far known to be generally upregulated in CRC and its metastases (41, 64), it is surprising that in our study EpCAM yielded the second highest fold change in CRLM-SD LM in comparison to the other LM subgroups. This finding underlines the heterogeneity of CRC-LM and emphasizes the significance of investigating both EpCAM levels and its function within CRC-LM tissues from different patients, rather than solely comparing tumor and metastatic tissue to normal tissues. Overall, based on the high abundance proteins in CRLM-SD compared to other LM tissue, the diagnosis of CRLM-SD-type LM in CRC patients is associated with cancer proliferation, increased risk of further metastases development, recurrence, and therefore poorer prognosis compared to CRLM-CA. This was reflected in the Kaplan–Meier analysis of patient OS (Fig. 5).

LM assigned to CRLM-CA on the other hand showed a completely different proteome profile regarding the enriched signaling pathways. The main pathway uniquely enriched in CRLM-CA LM was the classical complement system. With the complement system as one of the first lines of defense of the innate immune response, its alteration is strongly connected to a reduced proliferation or apoptosis of cancer cells (70). The comparison of complement proteins between different LM has not been discussed before although it seems to be highly important to understand the mechanistic reasons for this heterogeneity in CRC-LM. Although a tumor promotor effect for complement activation through the lectin pathway is known, the other ones seemed to be nonregulated in cancer especially CRC (71). In support of the theory of an antitumorigenic effect in CRLM-CA LM, we observed no elevated proteins necessary for an activation of the lectin pathway in CRLM-CA. Instead, those involved in the classical pathway were found to be more highly abundant. Pizarro-Bauerle et al. discovered an effective mechanism of hemocyanins, therapeutic glycoproteins that induce an antitumor immune response (51). They found that activation of the classical pathway occurs through C1 binding to immunoglobulins, leading to this antitumor response. Consequently, the upregulation of drivers of the classical complement pathway in CRLM-CA would be in line with the observed increased survival of patients in CRLM-CA-type CRC-LM. The Kaplan–Meier curve of CRLM-CA assigned LM reflects this thesis through enhanced OS of patients harboring this CRC-LM phenotype (Fig. 5). Furthermore, the fact that C1q is highly expressed in the stromal region of CRLM, as previously shown by Bulla *et al*., strongly supports a phenotype where complement activation is a hallmark of these CRLM that provides a survival advantage, rather than being a minor blood contamination of specimens (72). We also made a comparison of the relative abundance of the highly abundant blood proteins and found no clustering to a specific CRLM subgroup, suggesting the CRLM-CA type is not derived from blood contamination (Supplemental Fig. S4). Furthermore, we used IHC to stain for clusterin, a multifunctional protein relevant in the later part of the complement cascade. These findings were consistent with the LC-MS/MS analysis, that clusterin was only highly expressed in the CRLM-CA subgroup (Supplemental Fig. S5). Interestingly, in the CRLM-CA subgroup, clusterin was primarily observed in hepatocytes surrounding the tumor cells, highlighting an interaction between CRLM and surrounding tumor microenvironment. We note that sequencing studies report that clusterin is typically lower in expression in colorectal cancer than normal tissue (73), and the observation of longer OS of patients with the CRLM-CA phenotype suggests clusterin can also provide a cytoprotective role. We speculate that one way might be achieved by protection against MAC-mediated cytolysis (74, 75, 76), as we observed high levels of MAC associated complement proteins in the CRLM-CA subgroup. However, due to the limited access to multiple specimens per subgroup for IHC, further validation of the observed concentration differences in clusterin is necessary.

The proteome for CRLM-OM is vastly different from CRLM-SD and CRLM-CA and characterized by higher abundance of proteins involved in various liver-specific activities. A strong enrichment was especially observed in amino acid and fatty acid metabolism assigned pathways. In general, cancer cells are dependent on an active metabolism justified in their high energy consumption during proliferation (77). Consequently, an increased concentration of metabolic active enzymes in cancer is associated with faster tumor growth and poor disease outcome (77, 78). The fatty acid beta-oxidation proved its upregulation by higher abundance of most key enzymes in the metabolism circle.

The amino acid metabolism was found enriched in CRLM-OM and is known for a negative regulation in CRC (77). Not only do the various amino acid metabolic pathways provide energy in form of ATP for cancer cell proliferation and growth but also some amino acid metabolites like glutathione have the ability to regulate oxidative cell stress which in turn influences CRC development (79).

In summary, differential quantitative proteomics of CRC-LM have revealed three distinct groups of CRC-LM patients associated with distinct signaling and survival. The identified proteins may serve as new therapeutic targets and as biomarkers for a novel individualized treatment with respect to the observed subgroups. Moreover, we provided evidence that mass spectrometric proteomics analysis can be used in a multicenter approach including batch-effect correction to increase the cohort size and ensure the patient representation at an international level.

In the future, these signatures may be used for an improved risk stratification, prognosis prediction as well as identification of novel therapeutic targets. Also, further prospective clinical studies are required to evaluate the described subgroups regarding their clinical end points and to prove the prognosis prediction.

## Data Availability

The mass spectrometry proteomics data have been deposited to the ProteomeXchange Consortium via the PRIDE partner repository with the dataset identifier PXD055821. reviewer_pxd055821@ebi.ac.uk

## Supplemental Data

This article contains supplemental data.

## Conflict of Interest

The authors declare no conflict of interest.
