# Supplementary material for: Proteomic Landscape of Colorectal Cancer Derived Liver Metastasis Reveals Three Distinct Phenotypes With Specific Signaling and Enhanced Survival
Source: Mol Cell Proteomics. 2025 Jul 4;24(8):101026. doi: 10.1016/j.mcpro.2025.101026 (PMC12335997; doi:10.1016/j.mcpro.2025.101026)
Supplement: Supplementary Data 3 [file mmc3.docx]

***Supplementary Information***

**Proteomic landscape of colorectal cancer derived liver metastasis reveals three distinct phenotypes with specific signalling and enhanced survival**

Proteomic landscape of colorectal cancer liver metastasis: a multi-cohort study

**Authors: Paula Nissen^[1]^, Nadezhda V. Popova^[2]^, Antonia Gocke^[1],[3]^, Daniel J. Smit^[2],[4]^, Geoffrey Yuet Mun Wong^[5],[9]^, Matthew J. McKay^[9]^, Thomas J. Hugh^[5]^, Kerstin David^[6]^, Hartmut Juhl^[6]^, Hannah Voß^[1]^, Jens U. Marquardt^[7]^, Björn Nashan^[8]^, Hartmut Schlüter^[1]^, Mark P. Molloy^[9]^, Manfred Jücker^[2]^**

**Affiliations:**

**^[1]^** Section Mass Spectrometric Proteomics, University Medical Center Hamburg-Eppendorf, Hamburg, Germany.

**^[2]^** Institute of Biochemistry and Signal Transduction, University Medical Center Hamburg-Eppendorf, Hamburg, Germany.

**^[3]^** Center of Molecular Neurobiology (ZNMH), University Medical Center Hamburg-Eppendorf, Hamburg, Germany.

**^[4]^** Institute of Tumor Biology, University Medical Center Hamburg-Eppendorf, Hamburg, Germany

**^[5]^** Department of Upper Gastrointestinal Surgery, Royal North Shore Hospital, Sydney, NSW 2065, Australia.

**^[6]^** Indivumed GmbH, Hamburg, Germany

**^[7]^** Department of Medicine I, University Medical Center, Lübeck, Germany.

**^[8]^** Organ Transplantation Center, The First Affiliated Hospital of the University of Science and Technology of China, Hefei, 230001, China.

**^[9]^** Bowel Cancer and Biomarker Research Laboratory, Faculty of Medicine and Health, School of Medical Sciences, The University of Sydney, Sydney, NSW 2006, Australia.

**Supplementary Information:**

**Supplementary Tables (in excel file):**

Supplementary Table 1: CRC-LM Sample Overview

Supplementary Table 2: UKSH Raw Data

Supplementary Table 3: UKE Raw Data

Supplementary Table 4: Sydney Raw Data

Supplementary Table 5: Total Cohort Abundances

Supplementary Table 6: Number Identified Proteins Cohorts

Supplementary Table 7: GSEA Terms in each Group (in excel file)

Supplementary Table 8: IPA Terms enriched in each Group (in excel file)

Supplementary Table 9: Glycoproteins quantified in CRLM-CA CRC-LM (in excel file)

**Supplementary Figures:**

Supplementary Figure 1: Consensus Cluster Plus statistical output.

Supplementary Figure 2: Additional principal component analysis with batches, occurrence time and chemotherapy information annotated.

Supplementary Figure 3: Additional ANOVA-based hierarchical clustering with patient number, run order and additional clinical data annotated.

Supplementary Figure 4: Unsupervised hierarchical clustering of most abundant blood proteins and exemplary boxplots of three most abundant ones.

Supplementary Figure 5: Immunohistochemical staining of clusterin in FFPE tissue from three CRC-LM specimens annotated to the three CRC-LM subgroups and boxplot of the normalized protein abundance of clusterin in each CRC-LM phenotype.

**Supplementary Figures**

**Supp. Fig. 1:** Consensus Cluster Plus revealed a statistical optimal cluster number of three, visualized through the **A** PAC score with a minimum at three clusters using hierarchical clustering (hc) and k-means (km) algorithm. The consensus matrix for k=3 **B**, the CDF curve **C** and the delta area under the CDF curve **D** indicating that three groups is optimal for our cohort.

**Supp. Fig. 2:** Principal component analysis of all proteins identified in 70% of all samples is depicted as a scatter plot, with **A** batches, **B** time of liver metastases occurrence, and **C** whether the patient received chemotherapy before surgery or not.

**Supp. Fig. 3:** Heatmap visualization of Pearson-correlation based hierarchal clustering of all 1,667 ANOVA significant (q-value<0.05) proteins identified in 70% of all samples, with **A** Patient numbers annotated for each sample, and **B** run order of sample measurements together and additional clinical parameters annotated.

**Supp. Fig. 4:** **A** Unsupervised Pearson-correlation based hierarchical clustering of most abundant blood proteins identified in the dataset. Boxplots of normalized protein abundance of **B** haptoglobin, **C** hemoglobin and **D** human serum albumin for CRC-LM phenotypes assigned samples.

**Supp. Fig. 5:** **A** Boxplot displaying normalized protein abundance of clusterin in each CRC-LM phenotype. Immunohistochemical staining of clusterin in FFPE tissue of CRC-LM specimen assigned to **B** CRLM-SD phenotype, **C** CRLM-CA phenotype and **D** CRLM-OM phenotype.
